# Post Vaccination Guillain Barre Syndrome: A Case Report

**DOI:** 10.7759/cureus.2511

**Published:** 2018-04-20

**Authors:** Muhammad Wajih Ullah, Aisha Qaseem, Afshan Amray

**Affiliations:** 1 Cardiology, Mayo Clinic, Rochester, USA; 2 Internal Medicine, Ziauddin Medical University, Karachi, PAK; 3 Internal Medicine, Dow Medical College, Dow University of Health Sciences (duhs), Karachi, PAK

**Keywords:** rabies vaccination, preventive medicine, healthcare improvement, primary health, guillain-barré syndrome (gbs), post vaccination guillain-barre syndrome

## Abstract

Guillain–Barre syndrome is a rare but fatal autoimmune disease. The exact cause of Guillain–Barre syndrome is still unknown. The most common known etiology of Guillain–Barre syndrome is infectious disease notably caused by *Campylobacter jejuni*. A very small fraction of people can develop Guillain–Barre syndrome due to vaccines and vaccinations like a meningococcal vaccine, poliovirus vaccine, influenza vaccine, and rabies vaccine. Of all these, rabies is fatal invariably. It can be preventable if diagnosed early and post-exposure treatment is followed according to the World Health Organization guidelines. Older formulations of rabies vaccines are cultured in the neural tissues and have been found to have an increased risk of Guillain–Barre syndrome. Although less immunogenic older formulations of rabies vaccines are more commonly used in Asian and South American countries due to their cost-effective nature. There is little to no data available on the incidence of Guillain–Barre syndrome due to vaccinations in Pakistan. Most of the cases of Guillain–Barre syndrome due to vaccination are either undiagnosed or misdiagnosed. In this case report, we are presenting a case of vaccine-associated Guillain–Barre syndrome due to neural tissue anti-rabies vaccine in a young girl, who presented with lower limb weakness, inability to pass urine and abdominal pain.

## Introduction

Guillain–Barre syndrome is a rare fatal autoimmune disease. The exact cause of the disease is still unknown. In Pakistan and other developing countries, older formulations of rabies vaccines derived from the neural tissues are widely used because they are cost-effective. However, they are associated with higher incidence of neurological complications like Guillain–Barre syndrome, as compared to cell cultured vaccines, newer formulations, which have fewer side effects. In a tertiary care hospital of Pakistan, we witnessed a case of a young girl who developed lower limb weakness after 24 days of receiving neural tissue sheep brain anti-rabies vaccine.

## Case presentation

A 15-year-old girl presented to the emergency department with a history of progressive lower limbs weakness for 10 days, inability to pass urine and intermittent grade 6/10 lower abdominal pain for two days. She had no associated symptoms like fever, rash, headache, backache or blurring of vision. No antecedent respiratory tract infection or diarrheal illness. Past medical history was insignificant. About 24 days prior to these symptoms, she received neural tissue sheep brain anti-rabies vaccine following a dog bite. On physical examination, the patient was not in acute distress. She was afebrile and her vitals were: a) Blood pressure: 130/80 mm Hg; b) Respiratory rate: 18 breaths/minute; c) Heart rate: 102 bpm.

Neurological examination revealed no facial asymmetry and intact cranial nerves. Motor system examination of the lower limbs revealed following: a) Power of the knee: grade ⅕ below the knees; b) Power of the hips: grade ⅕ of the hip flexors and extensors bilaterally; c) Tone and reflexes of the legs: hypotonia of legs bilaterally with loss of knee and ankle reflexes bilaterally; d) Joint position and vibration sense: reduced joint position and vibration was noticed, and there was hyperesthesia of the soles bilaterally.

Upper limbs were normal on physical examination. The cardiorespiratory system was unremarkable on examination. On abdominal examination, urinary bladder was palpable above the symphysis pubis and was tender on palpation.

Laboratory investigations revealed hemoglobin of 12.5 g/dl with the hematocrit of 44%; TLC was 6500/cmm and ESR at the first hour using Westergren method was 23 mm. Cerebrospinal fluid examination (CSF) revealed clear fluid, normal opening pressure, glucose 75 mg/dl (normal range: 45–100 mg/dl), proteins 2.5 g/l (normal range: 0.18–0.45 g/dl) and WBCs 2/cmm (albuminocytologic dissociation).

Based on physical examination and CSF findings a provisional diagnosis of Guillain–Barre syndrome was made. Electrophysiological study (confirmatory test) revealed demyelinating polyneuropathy consistent with Guillain–Barre syndrome. Plasmapheresis and physical therapy sessions resulted in a nearly complete recovery in six to eight months.

## Discussion

Guillain–Barre syndrome is a rare but fatal autoimmune disease. It is characterized by progressive ascending paralysis with varying degrees of weakness, sensory abnormalities and autonomic dysfunction. Most patients with Guillain–Barre syndrome recover completely or nearly complete in about 8–10 months. One of the suggested mechanisms for the development of Guillain–Barre syndrome is molecular mimicry. An immune response triggered by a prior infection cross-reacts with the peripheral nerve components and mistakenly damaging their own myelin sheath [1]. Despite multiple proposed mechanisms behind the development of Guillain–Barre syndrome, the exact cause of Guillain–Barre syndrome is still unknown, and it may or may not have some triggering factor [2]. Among the triggering factors, vaccines, which include meningococcal vaccine, poliovirus vaccine, influenza vaccine, and rabies vaccine, are reported to be associated with the onset of Guillain–Barre syndrome [3].

Vaccine-associated Guillain–Barre syndrome is defined as those with the onset of Guillain–Barre syndrome symptoms within the six-week period after receiving the vaccine, as reported by Vaccine Adverse Event Reporting System (VAERS) [4]. Rabies is a fatal disease invariably. It can be preventable if diagnosed early and post-exposure treatment is followed according to the World Health Organization guidelines. The post-exposure treatment includes immediate vaccination, local treatment of the wound and passive immunization with rabies immunoglobulins [5]. The issue of post-exposure treatment of rabies is very concerning, as in Pakistan and other developing countries, neural tissue sheep brain anti-rabies vaccines are still being used despite the fact that they have low immunogenicity, more side effects and questionable efficacy [6]. Neural tissue sheep brain anti-rabies vaccines are associated with an increased risk for neurological complications like Guillain–Barre syndrome, with a reported incidence of about 1200 [7].

It is calculated that in Pakistan there are nearly 100,000 cases of dog bites, causing 5,000 deaths each year from rabies [8]. As the burden of this problem is already huge in Pakistan, most of the cases of Guillain–Barre syndrome due to vaccination are either undiagnosed or misdiagnosed. There is little to no data available on the incidence of Guillain–Barre syndrome due to vaccinations in Pakistan. However, there were few reported cases of Guillain–Barre syndrome following rabies vaccination from India [9].

## Conclusions

In Pakistan and other developing countries, neural tissue sheep brain anti-rabies vaccines are widely used because they are cost-effective. Neural tissue sheep brain anti-rabies vaccines are associated with higher incidence of neurological complications like Guillain–Barre syndrome in comparison with the cell culture vaccines which have fewer side effects. It is therefore recommended that use of neural tissue sheep brain anti-rabies vaccines should be condemned, and instead, the use of cell culture vaccines should be encouraged for the post-exposure treatment of rabies.
